# Ulcerated calcification of the interventricular septum causing Transient Ischemic Attacks: Case Report

**DOI:** 10.1186/1749-8090-2-19

**Published:** 2007-04-17

**Authors:** Matthew Panagiotou, Kostas Markakis, Nikolaos Mourtzis, Stella Economidis, James Crockett, Efstratios N Koletsis

**Affiliations:** 1 Department of Cardiac Surgery, Athens Medical Center, Athens, Greece; 2 Cardiothoracic Surgery Department, University of Patras, Greece

## Abstract

**Background:**

Calcific deposits are frequently observed at sites of healed myocardial infarcts. Grossly visible calcification of myocardial infarcts and calcified intracavitary cardiac thrombi are less common but recently are becoming more frequent findings during surgical ventricular restoration procedures.

**Case Presentation:**

A 64 years old male diabetic patient experienced two episodes of transient ischemic attacks during the last six months. During the diagnostic work up he was found to have triple vessel coronary artery disease with mild left ventricular dysfunction, akinesia of the anterior-apical wall and hypokinesia of the inferior wall. He was referred to our department for coronary artery bypass grafting. He underwent elective triple coronary artery bypass and a ventricular restoration procedure due to apical wall thinning. The inspection of the left ventricle revealed an ulcerated round shape calcification of the interventricular septum with a crater filled with clot. We resected the above lesion and covered the damaged area with the septal Dacron patch of the modified linear closure. The patient was discharged from the hospital on the 11^th ^postoperative day and has been doing well 6 months later, with improvement in both ventricular function and clinical status.

**Conclusion:**

The exploration of the left ventricular cavity reveals interesting phases of the post-infarction healing process. The suspicion of left ventricular thrombosis in patients with ventricular asynergy justifies a ventricular exploration during coronary artery bypass surgery.

## Background

Calcific deposits are frequently observed in human hearts. They are most commonly found in epicardial coronaries, mitral and aortic annular regions, apices of papillary muscles and at sites of healed myocardial infarcts [1]. Grossly visible calcification of myocardial infarcts, and calcified intracavitary cardiac thrombi, are becoming more frequent findings during surgical ventricular restoration procedures [2,3].

## Case report

A 64 years old male diabetic patient with coronary artery disease and a history of myocardial infarction had recently experienced two episodes of transient ischemic attacks. The presumed diagnosis was cerebral embolism since an intracranial hemorrhage was excluded and there was no detected ipsilateral carotid stenosis. The patient was in NYHA class III, with no angina. Thallium-201 myocardial scintigraphy showed a combination of scar and viable myocardium in the territory of the occluded left anterior descending.

On coronary angiography he was found to have triple vessel disease, with total occlusion of the left anterior descending branch and of the right coronary artery. Left ventriculography revealed left ventricular dysfunction with a LVEF of 35%, akinesia of the anterior-apical wall and hypokinesia of the inferior wall. He was referred to our department for coronary artery bypass grafting.

The operation was performed with the aid of cardiopulmonary bypass, moderate hypothermia and intermittent combined (retro and antegrade) cold blood cardioplegia. Intraoperatively the apical walls were found to be thin and scared and collapsed with ventricular venting.

**Figure 1:**
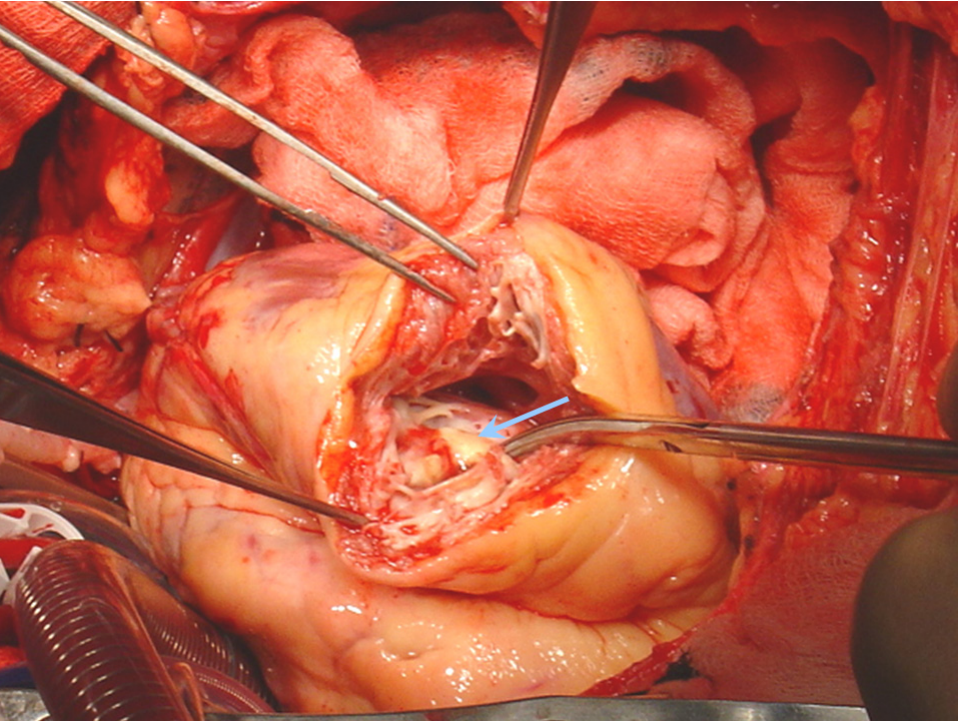
Intraoperative picture after opening the left ventricle. A round – shaped calcification (diameter of 5 cm) with a central crater (pale blue arrow) filled with clot was clearly evident at the peripheral interventricular septum.

We performed a triple coronary artery bypass with a left internal mammary artery to the intermediate artery (since it was the best target vessel) and two saphenous vein grafts to the obtuse marginal and the left anterior descending. The latter was endarterectomised due to chronic occlusion. Despite the fact that the patient had no absolute indication for a ventricular restoration (SVR) procedure, the extent of the damaged apical wall found intraoperatively and the history of the Transient Ischemic Attacks made us perform a ventriculotomy and an "anti-remodeling" procedure. After the ventriculotomy, inspection of the left ventricular cavity revealed a "round – shaped" calcification of the peripheral interventricular septum (diameter of 5 cm) with a central crater filled with clot (Figure 1).

**Figure 2:**
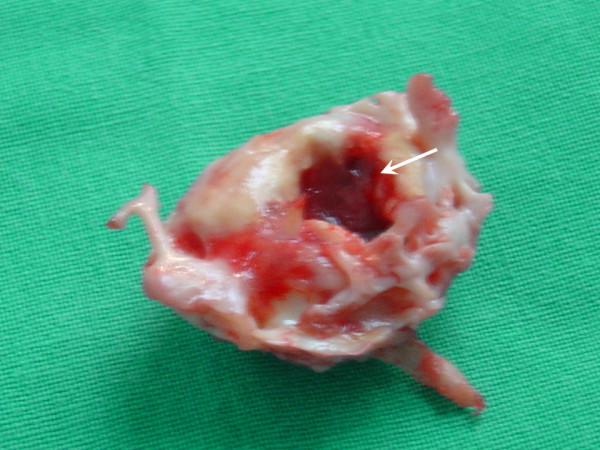
The round shaped calcification specimen with a central crater (white arrow) filled with clot.

We performed a resection of the found lesion together with the surrounding damaged endocardium (endocardiectomy) (Figure 2).

The area of the endocardiectomy together with the infracted peripheral septum was covered with a Dacron patch. Consequently this septal patch was incorporated in the linear closure of the anterior-apical ventriculotomy [4].

Postoperative recovery was uneventful. He was discharged from the hospital on the 11^th ^postoperative day and is doing well 6 months later, with improvement in both ventricular function (LVEF 45%) and clinical status (NYHA class I).

## Discussion

Left ventricular thrombus formation may occur in the early course after acute anterior myocardial infarction. Delayed thrombus formation is always associated with wall motion deterioration. Oral anticoagulant therapy is strongly recommended in these patients and associated with thrombus resolution and decrease of embolic events. Some times persistent LV mural thrombi may become encapsulated by thickened and calcified endocardium [5].

The inspection of the LV cavity performed during SVR procedures, reveals different stages of the post-infarction ventricular healing process, previously less well recognized.

The accepted indication for an SVR procedure according to the experience is a >35% asynergy of the left ventricular perimeter [6]. In cases with less extent of apical involvement but with significant wall thinning, a 'prophylactic' anti – remodeling muscle procedure is common practice [7]. Some surgeons are performing this procedure without even a ventriculotomy, by stitching 'blindly' the ventricular apex from outside. They define such a procedure as a minimally invasive one or an 'off-pump' ventricular restoration. The later procedure must be an absolute contraindication in cases with suspected ventricular thrombi, which in the author's experience are not rare even in patients on anticoagulation treatment.

## Conclusion

Inspection of the left ventricular cavity in ischemic cardiomyopathy patients with apical asynergy reveals interesting phases of the post-infarction healing process. Utilizing a lower decision threshold for a ventriculotomy and surgical ventricular restoration surgery can be advantageous especially in coronary patients with segmental asynergy and suspected ventricular thrombi.

## Abbreviations

**SVR: **Surgical Ventricular Restoration Procedure

**LV: **Left Ventricular

**LVEF: **Left Ventricular Ejection Fraction

## Competing interests

The author(s) declare that they have no competing interests.
